# CD38–Cyclic ADP-Ribose Signal System in Physiology, Biochemistry, and Pathophysiology

**DOI:** 10.3390/ijms23084306

**Published:** 2022-04-13

**Authors:** Shin Takasawa

**Affiliations:** Department of Biochemistry, Nara Medical University, 840 Shijo-cho, Kashihara 634-8521, Nara, Japan; shintksw@naramed-u.ac.jp; Tel.: +81-744-22-3051 (ext. 2227); Fax: +81-744-24-9525

**Keywords:** CD38, cADPR, RYR2, FKBP12.6, pancreatic β cells, CD157

## Abstract

Calcium (Ca^2+^) is a ubiquitous and fundamental signaling component that is utilized by cells to regulate a diverse range of cellular functions, such as insulin secretion from pancreatic β-cells of the islets of Langerhans. Cyclic ADP-ribose (cADPR), synthesized from NAD^+^ by ADP-ribosyl cyclase family proteins, such as the mammalian cluster of differentiation 38 (CD38), is important for intracellular Ca^2+^ mobilization for cell functioning. cADPR induces Ca^2+^ release from endoplasmic reticulum via the ryanodine receptor intracellular Ca^2+^ channel complex, in which the FK506-binding protein 12.6 works as a cADPR-binding regulatory protein. Recently, involvements of the CD38-cADPR signal system in several human diseases and animal models have been reported. This review describes the biochemical and molecular biological basis of the CD38-cADPR signal system and the diseases caused by its abnormalities.

## 1. Introduction

Changes in cytosolic Ca^2+^ are indispensable for normal cell function, such as secretion, proliferation, neuronal guidance, cell death, and development. Typically, cells respond to a Ca^2+^ signal that is generated inside the cell in response to the activation of a wide variety of extracellular signals, including those involved in nutrient, neurotransmitter, hormonal, and sensory signaling. In most cases, the initial Ca^2+^ signal generated in the cell is a specific increase in cytoplasmic Ca^2+^ concentrations resulting from the release of Ca^2+^ from internal Ca^2+^ stores (mainly the endoplasmic reticulum) or the entry of Ca^2+^ from the external spaces across the plasma membrane. Both routes involve the movement of Ca^2+^ through Ca^2+^ channels that are localized within these cellular membranes. While intracellular Ca^2+^ release from the endoplasmic reticulum occurs via channels activated by inositol 1,4,5-trisphosphate (IP_3_), cyclic ADP-ribose (cADPR), or Ca^2+^ itself (Ca^2+^-induced Ca^2+^ release), Ca^2+^ influx across the plasma membrane is achieved via numerous types of Ca^2+^ channels, including voltage-gated Ca^2+^ channels and store-operated Ca^2+^ channels as well as a variety of ligand-gated cation channels.

In this review, I focus specifically on the historical background, physiological significance, biochemical and molecular biological bases, and recent topics in the pathophysiology of the CD38-cADPR signal system in mammalian cells and human diseases.

## 2. cADPR as an Intracellular Ca^2+^-Releasing Messenger

Changes in cytosolic Ca^2+^ levels govern a multitude of cellular processes. Many extracellular stimuli mediate complex changes in Ca^2+^ through the production of second messengers [1]. These molecules effect the release of Ca^2+^ from the intracellular Ca^2+^ store. In addition to IP_3_, cADPR and nicotinic acid adenine dinucleotide phosphate (NAADP) play crucial roles in the generation of agonist-evoked Ca^2+^ signals [2,3]. IP_3_ is the best characterized of these molecules [1]. In addition, cADPR and NAADP, which were first discovered in sea urchin eggs [4], also play prominent roles in generating complex Ca^2+^ signals. cADPR induces Ca^2+^ release through the activation of the ryanodine receptors (RyRs) located on the endoplasmic reticulum [2,3].

Both cADPR and NAADP are synthesized by the same family of enzymes, the ADP-ribosyl cyclases (EC 3.2.2.6 and EC 2.4.99.20), which therefore play a central role in Ca^2+^ signaling and have been implicated in a variety of processes ranging from bacterial clearance to social behavior [5]. ADP-ribosyl cyclase (EC 3.2.2.6) activity was originally identified in sea urchin egg homogenates [4], but an enzyme with the activity was later purified [6,7] and cloned [8,9] from *Aplysia* ovotestes. The enzyme was shown to catalyze the cyclization of NAD^+^ to form cADPR and the replacement of the nicotinamide moiety in NADP with nicotinic acid to form NAADP. Based on amino acid sequence similarity, it became evident that the mammalian protein cluster of differentiation 38 (CD38; ADP-ribosyl cyclase 1; EC 3.2.2.6) [10,11,12,13] and cluster of differentiation 157 (CD157; ADP-ribosyl cyclase 2; EC 3.2.2.6) [14,15] were also ADP-ribosyl cyclase in mammalian cells.

## 3. cADPR in Insulin Secretion from Pancreatic β-Cells

**Figure 1:**
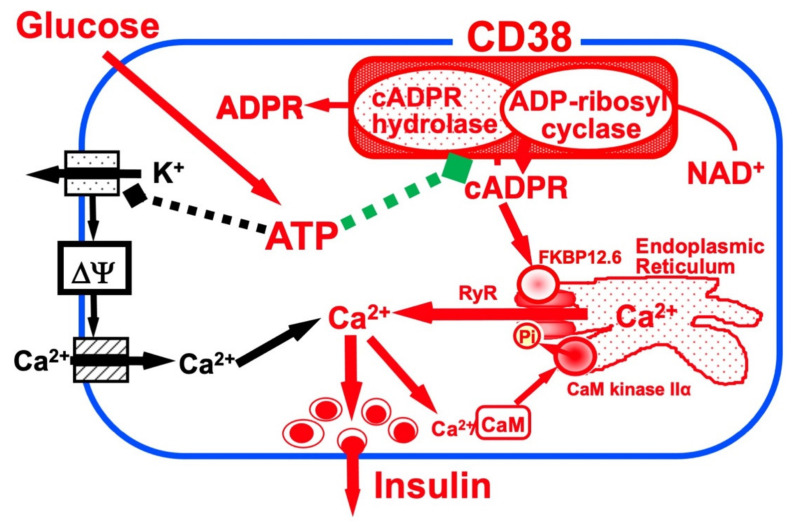
Insulin secretion by glucose stimulation in pancreatic β-cells (adapted from [33]). The insulin secretion via the CD38-cADPR signal system is shown in red. cADPR, produced from NAD^+^ by the ADP-ribosyl cyclase of CD38, binds to FKBP12.6 to release Ca^2+^, dissociating FKBP12.6 from RyR [25]. CaM kinase II phosphorylates RyR to sensitize and activate the Ca^2+^ channel (Pi, phosphorylation of RyR by CaM kinase II) [24]. Ca^2+^, released from intracellular stores and/or supplied from extracellular sources, further activates CaM kinase II and amplifies the process. In this way, Ca^2+^-induced Ca^2+^ release (CICR) can be explained in glucose-induced insulin secretion in pancreatic β-cells. The conventional insulin secretion mechanism by Ca^2+^ influx from extracellular sources [16] is shown in black.

Glucose induces an increase in intracellular Ca^2+^ concentration in pancreatic islet β-cells and induces the secretion of insulin. Ashcroft et al. first explained the importance of the increase in the Ca^2+^ concentration in 1984 [16]. The millimolar concentration of ATP is produced by the process of glucose metabolism in pancreatic β-cells. As a result, there is the closure of ATP-sensitive potassium (K_ATP_) channels, which results in membrane depolarization, the influx of Ca^2+^ through voltage-dependent Ca^2+^ channels, and an increase in the cytosolic Ca^2+^ concentration that triggers the exocytosis of insulin granules. In 1993, another model of insulin secretion by glucose via cADPR-mediated Ca^2+^ mobilization from an intracellular Ca^2+^ pool, the endoplasmic reticulum, was proposed [12,17], as shown in Figure 1. According to this model, ATP inhibits the cADPR hydrolase of CD38, causing the accumulation of cADPR, which acts as a second messenger for Ca^2+^ mobilization from the endoplasmic reticulum for insulin secretion [18,19,20,21,22,23,24,25,26,27,28,29,30,31,32,33,34].

The first important issue is whether the accumulation of cADPR is actually caused by glucose stimulation in pancreatic islets. In our laboratory, we assayed the cADPR content in islets isolated from normal rats (Wistar) and mice (C56BL/6J) that were incubated with low (2.8 mM) and high (20 mM) glucose by radioimmunoassay using an anti-cADPR antibody. The cADPR content in the high glucose-treated islets was rapidly increased within 5 min. In contrast, the cADPR content in the low glucose-treated islets was not [28].

Using the rat islet microsome Ca^2+^ releasing system, fluo 3 (2,2′-{[2-(2-{2-[Bis(caboxymethyl)amino]-5-}2,7-dichloro-6-hydroxy-3-oxo-3H-xanthen-9-yl)phenoxy]ethoxy}-4-methylphenyl]azanediyl}diacetic acid) fluorescence showed cADPR released Ca^2+^ [17,24,28]. IP_3_ did not cause the release of Ca^2+^, and after the addition of IP_3_, the islet microsomes were still responsive to cADPR. In contrast, IP_3_ caused a release of Ca^2+^ from cerebellum microsomes, and cADPR also caused a release of Ca^2+^. Heparin, an inhibitor of IP_3_ binding to its receptor, blocked the IP_3_-induced Ca^2+^ release from cerebellum microsomes but did not block the cADPR-induced Ca^2+^ release. These results indicate that islet microsomes respond to cADPR but not to IP_3_. In contrast, cerebellum microsomes respond to both cADPR and IP_3_, but cADPR induces the Ca^2+^ release via a different mechanism than that utilized by IP_3_.

The effect of cADPR on insulin secretion was examined using digitonin-permeabilized rat pancreatic islets. cADPR as well as Ca^2+^ induced insulin secretion, but IP_3_ did not. The combined addition of cADPR and Ca^2+^ did not induce significantly more insulin secretion than the addition of cADPR or Ca^2+^ alone. The cADPR-induced insulin secretion was inhibited by the addition of EGTA. These results indicated cADPR induced Ca^2+^ release from islet microsomes and the increment of Ca^2+^ resulted in insulin secretion from pancreatic islets [17].

From these results, glucose-induced insulin secretion via cADPR formation from NAD^+^ and cADPR-induced Ca^2+^ mobilization from the endoplasmic reticulum was proposed. Concerning the second messenger role of cADPR for glucose-induced insulin secretion, some researchers reported that cADPR-induced intracellular Ca^2+^ release was not observed in *ob/ob* mouse islets and rat insulinoma-derived RINm5F cells [19,35,36,37,38]. Malaisse et al. [39,40] measured the cADPR content in rat islets and reported that it appeared not to be significantly affected by glucose concentrations. On the other hand, the fasting of the rats before the isolation of islets and the usage of Hanks’ solution containing 2.8 mM glucose during the islet isolation may account for the rapid and significant increase in cADPR content in the islets in response to glucose stimulation. Furthermore we determined the cADPR content by assessing the recovery of cADPR in the extraction and concentration procedures, and they did not. Differences in the experimental conditions may be responsible for the different results [19,28]. Nevertheless, the reports that Balb/c mouse islets showed increases in glucose-induced cADPR production, the intracellular concentration of Ca^2+^, and insulin secretion [41] and that human insulin promoter-SV40 large T transgene-introduced C57BL/6 mouse pancreatic β-cell-derived MIN6 cells, which show glucose-induced insulin secretion, showed a dramatic Ca^2+^ mobilization in response to cADPR via the ryanodine receptor (RyR) [42,43] despite the lack of response to IP_3_ [43]. These results indicate that the CD38-cADPR signal system is functioning in glucose-induced insulin secretion in pancreatic β-cells.

## 4. CD38 as a Major Enzyme for the Synthesis of cADPR

**Figure 2:**
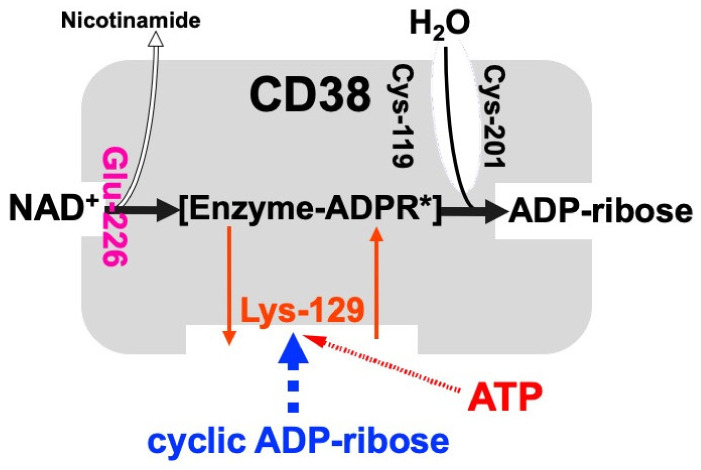
Schematic representation of human CD38 in enzyme activities for the synthesis and hydrolysis of cADPR (adapted from [26]). Glu-226 in human CD38 is essential for cADPR synthesis from NAD^+^ (ADP-ribosyl cyclase activity) [46]. Cys-119 and Cys-201 are essential for cADPR hydrolysis to form ADP-ribose (cADPR hydrolase activity). Lys-129 is the cADPR binding site and is indispensable for cADPR hydrolysis (cADPR hydrolase) [21]. ATP, produced by glucose metabolism, competes with cADPR for the binding site (Lys-129), inhibiting the cADPR hydrolysis by cADPR hydrolase of CD38, which causes the accumulation of cADPR in the cell [12]. [Enzyme-cADPR*] is proposed as an enzyme-stabilized ADP-ribosyl oxocarbonium ion intermediate.

CD38 is a 300-amino-acid protein and was first recognized as a human leukocyte surface antigen. CD38 was found to express in a variety of tissues and cells, including pancreatic β-cells [12,20,44]. We and others have found that CD38 has both ADP-ribosyl cyclase for cADPR synthesis from NAD^+^ and cADPR hydrolase for the hydrolysis of cADPR to form ADP-ribose [11,12,13,21,45]. Using purified human CD38 protein, it was found that millimolar concentrations of ATP inhibit the cADPR hydrolase activity of CD38, competing with the substrate, cADPR [12,26]. The competitive inhibition of the cADPR hydrolysis by ATP suggests that ATP and cADPR bind to the same site of CD38. We purified human recombinant CD38, incubated with an ATP analogue, 5′-p-fluorosulfonylbenzoyladenosine, and identified the binding site for ATP and/or cADPR as the lysine-129 of CD38 [26]. From these results and other available evidence, we proposed that CD38 catalyzes the formation of cADPR from NAD^+^ and also the hydrolysis of the cADPR to ADP-ribose. As shown in Figure 2, lysine-129 of CD38 is the cADPR binding site for hydrolysis to ADP-ribose and ATP competes with cADPR in the site, resulting in the inhibition of the hydrolysis of cADPR and then in the accumulation of cADPR by ATP [12,21,34].

**Figure 3:**
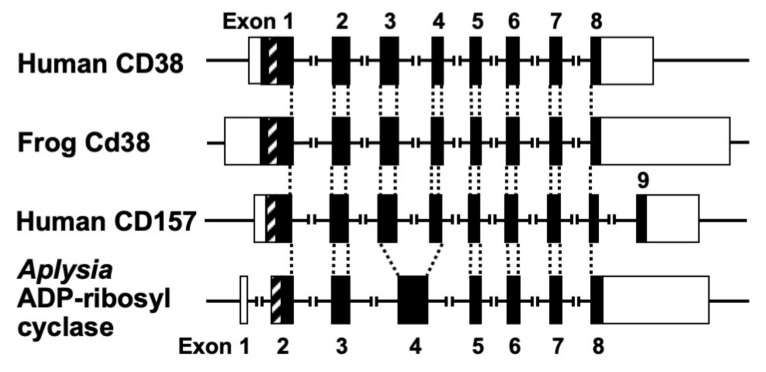
Structural organization of human CD38, frog Cd38, human CD157, and *Aplysia kurodai* ADP-ribosyl cyclase genes (adapted from [9,27,44]). The exons are depicted as boxes; filled and open boxes represent protein-coding regions and untranslated regions, respectively. Recently, primate-specific exon 1b, located between exon 1 and 2 of human CD157, that encodes 15 additional amino acids, was reported [48]. Hatched boxes represent transmembrane (CD38)- or signal peptide (CD157 and *Aplysia* ADP-ribosyl cyclase)-coding regions. The corresponding exon-intron junctions are indicated as broken lines. Exons are numbered.

The human *CD38* gene was assigned to band p15 of chromosome four as a single copy gene by in situ fluorescence hybridization [22]. We then isolated the human *CD38* gene and determined its primary structure [27]. The human *CD38* gene as well as the *Xenopus Cd38* gene extending ~70 kb [27,44] contained eight exons (Figure 3). The human and *Xenopus Cd38* promoter regions have no TATA box but do have a CpG island, a methylation-controlled region more frequently associated with housekeeping than tissue-specific genes. Exon one encoded the 5′-untranslated region, translational start site, putative transmembrane domain and N-terminal region of Cd38. Exons two to eight encoded the remainder of Cd38. Cycsteines-119 and -201 of human CD38, which are indispensable for the hydrolysis of cADPR to form non-Ca^2+^ mobilizing ADPR [21], were encoded in exons two and five, respectively. Lysine-129, which is involved in cADPR binding to CD38 [26], was encoded in exon three. Glutamate-226 and Trp-185, which seem to play important roles in catalytic activities [46,47], were encoded in exons six and four, respectively. The 10 cysteine residues conserved among human, rat, mouse, chicken, and frog Cd38s; human, rat, mouse, chicken, and frog Cd157s; *Aplysia* ADP-ribosyl cyclases, purple sea urchin ADP-ribosyl cyclase, and blood fluke ADP-ribosyl cyclase were encoded into six exons [27,32,44]. The translational termination codon and 3′-untranslated region were located in exon eight.

Isolation and determination of the primary structure of the *Aplysia kurodai ADP-ribosyl cyclase* gene [9] and the frog (*Xenopus laevis*) *Cd38* gene [44] as well as the human *CD38* gene [27] were performed. The exon–intron organization of the *Aplysia* ADP-ribosyl cyclase gene and the frog *Cd38* gene is very similar to that of the human *CD38* gene, suggesting that they evolved from a common ancestral gene [27,44] (Figure 3).

**Figure 4:**
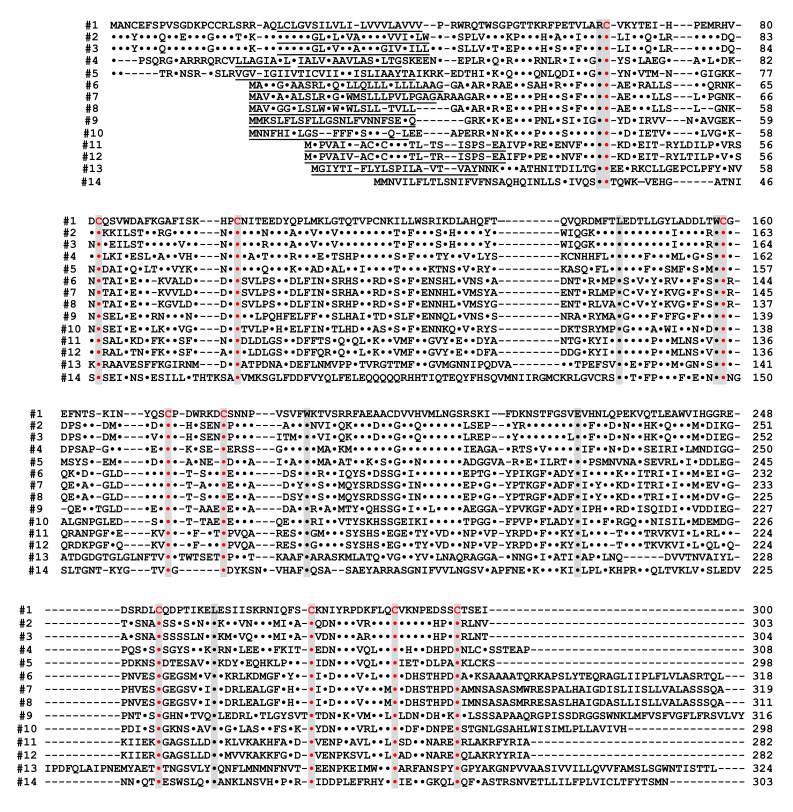
Alignment of the amino acid sequences of the CD38/CD157/ADP-ribosyl cyclase family based on the primary structures of the encoded proteins (adapted from [44]). Dots indicate amino acids identical to human CD38. Dashes indicate gaps for maximal alignment. Ten cysteine and five amino acid residues conserved among all the proteins are highlighted in red and gray, respectively. The underlined amino acid residues are a putative transmembrane domain in CD38 and signal sequence in CD157 and ADP-ribosyl cyclase. The sequences used in alignment are #1: human CD38 (AAA68482 [55]), #2: rat CD38 (BAA06129 [20]), #3: mouse CD38 (AAA03163 [56]), #4: chicken CD38 (NM_001201388), #5: *Xenopus laevis* CD38 (AB194899 [44]), #6: human BST1 (CD157) (BAA04885 [47]), #7: rat BST1 (CD157) (Q63072 [51]), #8: mouse BST1 (CD157) (BAA06597 [57]), #9: chicken CD157 (NM_001200043), #10: *Xenopus laevis* CD157 (AB194901 [44]), #11: *Aplysia californica* ADP-ribosyl cyclase (AAA65698 [8]), #12: *Aplysia kurodai* ADP-ribosyl cyclase (BAA06284 [9]), #13: *Strongylocentrotus purpuratus* ADP-ribosyl cyclase (AM494973 [58]), and #14: *Schistoma mansoni* ADP-ribosyl cyclase (AAX35328 [59]). Although XP_005162637 (*Danio rerio* ADP-ribosyl cyclase 1-like [60]) showed significant homology with CD38/CD157/ADP-ribosyl cyclase family, the 8th and 10th cysteines in the conserved 10 cysteine residues (red), which are essential for the enzyme activities, are not conserved. It may be important to measure ADP-ribosyl cyclase activity in zebrafish ADP-ribosyl cyclase 1-like for understanding the involvement of the two cysteines in ADP-ribosyl cyclase activity.

Kaisho et al. [49] found that the amino acid sequence of bone marrow stromal antigen 1 (BST-1/CD157) had significant sequence homology (~30% identity) with those of *CD38* and *Aplysia* ADP-ribosyl cyclase. They determined the gene structure of human *CD157* [50]. The gene extended for ~30 kb, very close to its paralogue *CD38*, and consisted of nine exons. Comparison of the gene organization reveals that the human *CD157* gene has an exon–intron organization similar to that of human *CD38* and *Aplysia* ADP-ribosyl cyclase genes (Figure 3). However, the human *CD157* gene has an additional exon, exon nine, that encodes a peptide that is removed upon attachment of the glycosyl phosphatidylinositol anchor. In addition to the similar exon–intron structures of CD38, CD157 and *Aplysia* ADP-ribosyl cyclase genes and human CD38 and CD157 genes are located on the subregion of the human chromosome 4p15 as a next neighbor; the fact that mouse *Cd157* (known as BP-3) is very close to the map site of the *Cd38* gene on mouse chromosome 5 [51] strongly suggests that the three genes (*CD38*, *CD157*, and *Aplysia* ADP-ribosyl cyclase) have evolved from a common ancestor, and *CD38* and *CD157* genes were created by gene duplication before human and rodent divergence (Figure 4).

The expression of Cd157 was reported in rat and mouse pancreatic β-cells [52,53]. However, no diabetic features were observed in Cd157 knockout mice [54]. Furthermore, CD157 exhibits neither cADPR-synthesizing (ADP-ribosyl cyclase) nor -hydrolyzing (cADPR hydrolase) activity in physiological conditions [14], suggesting that CD157 may mainly play a role as a surface antigen rather than a major enzyme for the synthesis of cADPR in response to glucose stimulation in pancreatic β-cells.

## 5. Physiological Significance of the CD38-cADPR Signal System in Mammalian Cells

In order to confirm the proposed mechanism of insulin secretion, in which the CD38-cADPR signal system plays a major role, CD38 transgenic, CD38 knockout, and FKBP12.6 knockout mice were produced [23,31,61]. In 19995, transgenic mice overexpressing human CD38 in pancreatic β-cells using rat insulin II promoter were produced [23]. Using immunohistochemistry, we demonstrated that the pancreatic islets of the two independent transgenic lines were densely stained for human CD38 [23]. The insulin secretion from the transgenic islets was significantly higher than that from the control islets at high concentrations of glucose. When islets were exposed to α-ketoisocaproate (4-methyl-2-ozopentanic acid), which, like glucose, is metabolized to form ATP, the insulin secretion from the transgenic islets was significantly higher than the control. However, with tolbutamide and KCl, the transgenic insulin secretion was not altered as compared to the control. Tolbutamide blocks the ATP-sensitive K^+^ channels, and KCl directly induces cell membrane depolarization resulting in Ca^2+^ influx from extracellular space [62]. In glucose-tolerance tests using living animals, the serum insulin level of CD38-transgenic mice in response to glucose charange was higher than that of control mice.

Furthermore, CD38 gene-disrupted mice were created [31]. CD38 knockout mice developed normally but showed no increase in their glucose-induced production of cADPR in pancreatic islets. Both the glucose-induced rise in intracellular Ca^2+^ concentration and insulin secretion were severely impaired in CD38 knockout islets, whereas CD38 knockout islets seemed normal compared to the typical extracellular Ca^2+^ influx stimulants such as tolbutamide and KCl. CD38 knockout mice showed impaired glucose tolerance, and the serum insulin level in response to glucose stimulation was significantly lower than the control. In an insulin tolerance test, the blood glucose levels of CD38 knockout mice 15–60 min after insulin injection were essentially similar to those of wild-type mice, suggesting that the glucose intolerance found in CD38 knockout mice is the result of an impaired glucose-induced insulin secretion rather than an increase in peripheral insulin resistance. Further experiments were performed regarding whether the observed phenotype (reduction in glucose-induced insulin secretion) could be rescued by the pancreatic β-cell-specific expression of human CD38 cDNA. Transgenic mice carrying a human CD38 cDNA under the control of the rat insulin II promoter described above were crossed with CD38 knockout mice. CD38 knockout mice carrying the human CD38 transgene were generated by intercrossing between CD38 knockout and CD38 transgenic mice. The resultant CD38 knockout mice having human CD38 transgene ameliorated the glucose intolerance and the decreased insulin secretion observed in CD38 knockout mice, suggesting that the observed phenotype in CD38 knockout mice is indeed caused by the disruption of the CD38 gene in pancreatic β-cells [31]. Thus, these results using CD38-transgenic and CD38 gene-disrupted mice provide further evidence for the contribution of the CD38-cADPR signal system to the glucose-induced Ca^2+^ release from intracellular Ca^2+^ stores for insulin secretion.

**Figure 5:**
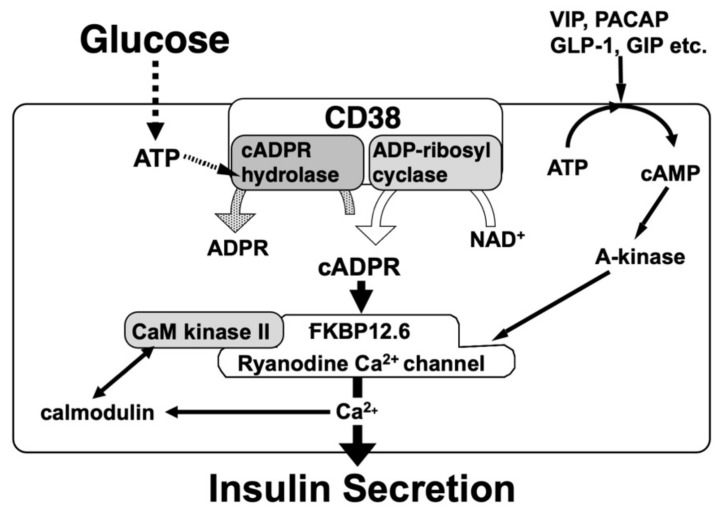
Possible model for glucose-induced insulin secretion via the CD38-cADPR signal system and its enhancement by CaM kinase II and A-kinase. CaM kinase II and A-kinase phosphorylate and activate RyR in pancreatic β-cells to secrete insulin much more. Peptide hormones such as vasoactive intestinal peptide (VIP), pituitary adenylate cyclase-activating peptide (PACAP), glucagon-like peptide-1 (GLP-1), and glucose-dependent insulinotropic polypeptide/gastric inhibitory polypeptide (GIP) activate adenylate cyclase (A-kinase) to increase cAMP. As a result, RyR Ca^2+^ channel is phosphorylated and sensitized/activated for cADPR-induced Ca^2+^ release.

It is currently accepted that cADPR activates a ryanodine receptor (RyR) Ca^2+^ channel to release Ca^2+^ from the endoplasmic reticulum [63]. Type 2 RyR (RyR2) expression in rat pancreatic islets was confirmed [19,64]. Experiments using rat islet microsomes revealed that cADPR did not bind directly to the RyR channel but acted on the RyR channel through a mediator, such as the FK506 (tacrolimus)-binding protein 12.6 (FKBP12.6), to release Ca^2+^. The cellular target for FK506, one of the most widely used immunosuppressive agents in clinical situations, are FKBP12 (peptidyl-prolyl cis-trans isomerase FKBP1A, immunophilin FKBP12) and FKBP12.6 (peptidyl-prolyl cis-trans isomerase 1B, immunophilin FKBP12.6). Rat FKBP12 is 108-amino-acid protein and is highly conserved among human, mouse, bovine, and rabbit FKBP12. Rat FKBP12.6 is also a 108-amino-acid protein, and the amino acid sequence is completely conserved in human and bovine FKBP12.6. Noguchi et al. [25] isolated microsomes from rat islets and found that rat islet microsomes contained FKBP12.6 but did not contain FKBP12 by Western blot analysis. A binding experiment and its Scatchard plot analysis revealed that cADPR binds to FKBP12.6 at a *Kd* value of 35 nM. The cADPR-binding to FKBP12.6 was inhibited by FK506, but neither structurally nor functionally related analogues of cADPR (NAD^+^, ATP, ADPR, nicotinamide, IP_3_, and ryanodine) attenuated cADPR binding to FKBP12.6. These results indicate that FKBP12.6 functions as a cADPR-binding protein and strongly suggest that cADPR is a physiological ligand for FKBP12.6, since FK506 does not naturally exist in mammalian cells except for the use of FK506 as an immune-suppressant/immune-modulator. Noguchi et al. prepared rat microsomes and treated them with cADPR and reported that FKBP12.6, occurring in rat microsomes, was dissociated from the microsomes, releasing Ca^2+^ from them. When the microsomes had been treated with FK506 or cADPR, FKBP12.6 dissociated from the microsomes and the FKBP12.6-dissociated microsomes did not show Ca^2+^ release in response to cADPR. FKBP12.6, as a component of RyR-FKBP12.6 complex Ca^2+^ channel, binds to cADPR and dissociates from the complex and the RyR channel activity is increased to release Ca^2+^. Therefore, cADPR cannot act on the RyR complex to release Ca^2+^ in the presence of FKBP12.6, and the glucose-induced insulin secretion does not function. In fact, Noguchi et al. made FKBP12.6-deficient mice that showed glucose intolerance coupled to insufficient insulin secretion upon glucose challenge. Insulin secretion in response to glucose was markedly impaired in FKBP12.6-deficient mouse islets, while sulfonylurea- or KCl-induced insulin secretion was unaffected, indicating that FKBP12.6 plays a role in glucose-induced insulin secretion downstream of ATP production, independently of ATP-sensitive K^+^ channels, in pancreatic β-cells [61]. In the clinic, when FK506 (tacrolimus) was used as an immunosuppressant agent in kidney transplantation, hyperglycemia was observed in 20–35% of the transplantation recipients [65]. This diabetogenic side effect of FK506 in transplantation may be explained by the mechanism shown in Figure 1. It should also be noted that the islet microsome did not contain calmodulin (CaM), islet microsomes became sensitized to cADPR at much lower concentrations for Ca^2+^ release in the presence of CaM, and the Ca^2+^ release was greatly increased [24]. Inhibitors for CaM and CaM kinase II (W7, KN62, and KN-93) completely abolished the glucose-induced insulin secretion as well as the cADPR-mediated and CaM-activated Ca^2+^ mobilization, but non-inhibitory analogues (W5, KN-04, and KN-92). Furthermore, autocamtide-2-related inhibitory peptide (called as AIP), which is more specific CaM kinase II peptide inhibitor, inhibited the activation of cADPR-mediated Ca^2+^ release from islet microsomes. Immunoblot analyses revealed that rat microsomes contained CaM kinase IIα but did not contain CaM. When the active 30 kDa chymotryptic fragment of CaM kinase II, which lacks the autoinhibitory domain of CaM kinase II and is therefore activated in the absence of CaM, was added to the microsomes, fully activated cADPR-mediated Ca^2+^ release was observed in the absence of CaM [24]. These results suggest that CaM kinase II is required to phosphorylate and activate the RyR for the cADPR-mediated Ca^2+^ release. Cyclic AMP (cAMP)-dependent protein kinase (A-kinase) activated the cADPR-mediated Ca^2+^ release from islet microsomes [33]. In the absence of A-kinase, only a small amount of Ca^2+^ was released from the microsomes by low concentrations of cADPR. Alternatively, when the catalytic subunit of A-kinase was added to the islet microsome Ca2+ release system, the Ca^2+^ release from the microsomes was sensitized at much lower concentrations of cADPR [33]. As incretin peptide hormones, such as glucagon-like peptide-1 (GLP-1) and gastric inhibitory polypeptide (GIP, also known as glucose-dependent insulinotropic polypeptide), increase the intracellular cAMP concentrations to activate A-kinase and exchange protein activated by cAMP2 (EPAC2)/cAMP-guanine nucleotide exchange factor II [66], the cADPR-mediated Ca^2+^ mobilization for insulin secretion could be activated via the phosphorylation of RyR by CaM kinase II and/or A-kinase/EPAC2. Possibly, the activated kinases phosphorylate the RyR channel to sensitize the cADPR signal (Figure 5). Dr. Kim and his co-workers showed the role of cADPR in the insulin secretion by GLP-1 [67].

**Figure 6:**
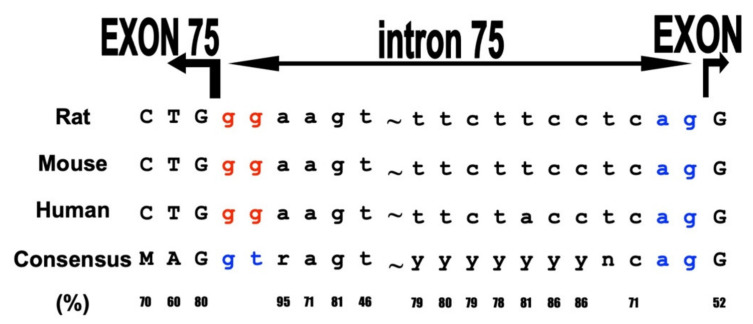
Usage of “gg” in splice donor sites of rat, mouse, and human *RyR2* intron 75 (adapted from [64,68]). RNA splicing in eukaryote follows the “GT-AG” rule by both cis-elements and regulatory trans-acting factors. Donor site dinucleotides of *RyR2* intron 75 in rat, mouse, and human are “gg” but not consensus “gt.” In consensus, “M”, “r”, “y”, and “n” represent A or C, a or c, c or t, and a, c, g, or t, respectively.

The CD38-cADPR signal system consists of two parts, the cADPR synthesis and metabolism, in which CD38 is a main player, and the Ca^2+^ release in response to cADPR from the RyR intracellular Ca^2+^ channel. There are three types of RyR in mammalian cells (type one, formerly called “skeletal type”), RyR2 (type two, formerly called “cardiac type”), and RyR3 (type three, formerly called “brain type”). The three types of RyR (RyR1, RyR2, and RyR3) are encoded by three unique genes and located in distinct chromosomal regions (19q13.2, 1q43, and 15q13.3-q14 in the human chromosome, respectively). RyR1 and RyR2 are predominantly expressed in skeletal muscles and cardiac muscles, respectively, and the names “skeletal type” and “cardiac type” were derived from the expressed tissues. In contrast, RyR3 was first isolated from brain and is now understood to express ubiquitously, including in the brain. Despite extensive physiological/pharmacological studies, the type of RyR that is the target for cADPR remains elusive. RyR2 appears to be expressed in pancreatic islets [19,28,63]. In order to determine the target RyR in glucose-induced insulin secretion from pancreatic β-cells, we screened the rat islet cDNA library and isolated a RyR cDNA clone using all the types of RyR cDNA as probe(s) and found it a novel RyR (islet-type RyR) that is generated from the *RyR2 gene* by the alternative splicing of exons 4 and 75 [64]. The deduced protein of the rat *RyR2* cDNA isolated from islet cDNA library was 4947 amino acids with a calculated molecular weight of 562,291 daltons. These two regions, corresponding to the seven amino acids in exon 4 and the twelve amino acids in exon 75 of the authentic cardiac *RyR2* gene, were not found in the *RyR2* cDNA isolated from the rat islet cDNA library. Interestingly, neither exon 4 nor exon 75 were found in human and mouse *RyR2* mRNA expressed in islets [19,64]. Further analyses of the alternative splicing patterns of *RyR2* mRNA expressed in several rat and mouse tissues revealed that the islet-type *RyR2* (lacking exons 4 and 75) was expressed not only in islets but also in other tissues such as cerebrum, cerebellum, pituitary gland, and adrenal gland. The cardiac-type (authentic) *RyR2* was expressed in the atrium, ventriculum, and kidney. In many other tissues, such as those of the jejunum, ileum, colon, and liver, both types of *RyR2* mRNA are expressed. In the human *RYR2* gene, the tissue-specific alternative splicing pattern was essentially similar to that of the rat and mouse gene (see Figure 6), and the islet-type *RYR2* mRNA (lacking exons 4 and 75) was also expressed in the human brain as well as in islets [19,64]. Interestingly, the splice donor consensus “gt” was substituted by “gg” in the intron 75 (1.0 kbp and 0.8 kbp introns in rat and human *RyR2* genes, respectively), whereas all the other splice donor and acceptor sequences conformed to the consensus “gt/ag rule” for splicing [19,64].

By RT-PCR analyses of human and mouse RNAs, the expression of (alternatively spliced) pancreatic islet-type *RyR2* mRNA was found not only in rat but also in human and mouse pancreatic islet RNAs. Moreover, the human and mouse islet-type *RyR* mRNAs expressed in islets and neuro-endocrine cells were also generated from the *RyR2* gene by alternative splicing. The splice donor consensus “gt” was also substituted by “gg”. As shown in Figure 6, intron 75 of the *RyR2* gene was spliced using “gg” as a donor for splicing instead of canonical “gt” in generating rat, mouse, and human authentic *RyR2* mRNAs. The *RyR2* gene for the CD38-cADPR signal system produced two different messenger RNAs by alternative splicing; one is for pancreatic islet β-cells and neuro-endocrine cells using canonical “gt/ag” splicing, and the other is for heart and blood vessels using not only the canonical “gt/ag” intron splicing site but also using the novel “**gg**/ag” site. Therefore, the heart/blood type RyR2 can contain exon 4 and exon 75 (Figure 6).

The islet-type RyR showed a greater increase in the Ca^2+^ release by caffeine when the RyR2-expression vectors (islet-type RyR2 and cardiac-type RyR2) were introduced and expressed in HEK293 cells pretreated with cADPR, suggesting that the novel islet-type RyR was an intracellular target for the CD38-cADPR signal system in mammalian cells, playing many important physiological roles in the functioning of the cADPR-sensitive Ca^2+^ release [64,68]. Most recently, it was reported that the replacement of “gg” with “gt” in intron 75 of the human insulin gene by gene-editing technology resulted not only in the reduction in glucose-induced insulin secretion but also glucose-induced proinsulin biosynthesis in 1.1B4 human insulin-producing cells [69], indicating that islets-type *RyR2* was essential for insulin biosynthesis via Ca^2+^ homeostasis. The use of “gg” as a splice donor has not been reported in disease-free conditions [70,71]. The utilization of the “gg” as a splice donor was only reported in the cholesteryl ester transfer protein gene in a patient with cholesteryl ester protein deficiency [72].

## 6. Pathological Significance of the CD38-cADPR Signal System

### 6.1. Pathological Significance of the CD38-cADPR System in Diabetes

Yagui et al. analyzed the CD38 gene in 31 Japanese type 2 diabetes patients who had first and/or second-degree relative(s) with type 2 diabetes by single-stranded conformation polymorphism [29]. Two variant patterns were noted in exons three and four of the CD38 gene. Sequence analysis showed that one of the variants in exon four was a silent mutation in the codon for Ile-168 of CD38 from ATA to ATC. The second change in exon three resulted in amino acid substitution, in which Arg-140 (CGG) was replaced by Trp (TGG). Yagui et al. further studied the two variants in 90 non-diabetic controls who had no family history of diabetes and showed normal glucose tolerance. The frequency of the Ilw-168 (ATC) allele was 16% in type 2 diabetes patients as well as in controls. However, the Trp-140 (TGG) allele was observed in 4 of 31 type 2 diabetes patients but not at all in any controls. When the Arg140Trp mutant protein was expressed in COS-7 cells, both ADP-ribosyl cyclase and cADPR hydrolase activities were reduced to 40–50% of the normal CD38 enzymic activity. The decreased function of the CD38 mutant may have contributed to the impairment of glucose-stimulated insulin secretion in type 2 diabetes patients.

**Figure 7:**
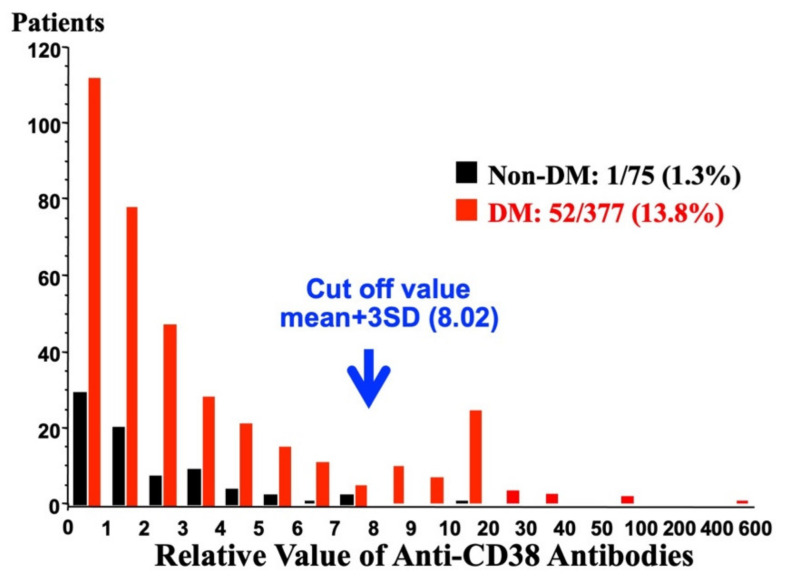
Distribution of relative values of anti-CD38 autoantibodies in 377 Japanese diabetic patients (adapted from [30]). The relative levels of CD38 antibodies are expressed as an index value relative to a standard non-diabetic serum. Values above the mean + 3 SD of the control samples (non-DM) were regarded as antibody positive. The arrow indicates a mean + 3 SD of non-diabetic control. Anti-CD38 positive patients were observed in diabetic patients.

A monoclonal antibody against CD38 (T16) was reported to inhibit the enzymic activity of CD38 [12]. The antibody significantly inhibited the glucose-induced insulin secretion [30]. Therefore, when anti-CD38 antibodies are present in patients, glucose-induced insulin secretion could be impaired and result in type 2 diabetes. Ikehata et al. screened the existence of anti-CD38 antibodies in Japanese diabetes patients by Western blotting and found some type 2 diabetic sera showed a positive reaction with recombinant human CD38 [30]. When the diabetic serum was incubated with recombinant CD38 to absorb anti-CD38 autoantibodies, the signals in Western blot analysis were abolished, indicating that they contained anti-CD38 autoantibodies. The screening of sera for autoantibodies against CD38 was performed in 377 Japanese type 2 diabetes patients and 75 non-diabetic controls who had no family history of diabetes and showed normal fasting plasma glucose levels. In non-diabetic subjects, the relative antibody values were within the mean + 3 standard deviation (SD) of non-diabetic subjects except in one subject. In contrast, the distribution of relative anti-CD38 values showed two clear peaks in type 2 patients: one was below the mean + 3 SD of non-diabetic controls and the other was over the mean + 3 SD (Figure 7). Thus, Ikehata et al., set the cut of value as mean + 3 SD (8.02) of non-diabetic controls and revealed that 52 of 377 (13.8%) patients with type 2 diabetes had anti-CD38 autoantibodies [30].

Although the general characteristics such as gender, age, duration of diabetes, body mas index, fasting plasma glucose, HbA1c, and treatment of diabetes (diet, oral agents, or insulin) did not show significant differences between the anti-CD38 autoantibody positive and negative type 2 diabetes patients, there was an inverse correlation between the age of onset of diabetes and the relative value of the anti-CD38 autoantibody (*p* = 0.0002, *r* = −0.513) [30]. Of the 52 anti-CD38 autoantibody positive type 2 diabetic sera, seven diabetic sera showed a positive reaction for recombinant rat CD38. The examination of the effects of the diabetic sera that showed a positive reaction to rat CD38 on glucose-induced insulin secretion from isolated rat islets was carried out. All the seven diabetic sera with a positive reaction to recombinant rat CD38 significantly and dose-dependently inhibited glucose-induced insulin secretion from isolated rat islets, whereas the control sera did not show such an inhibitory effect on the glucose-induced insulin secretion [30]. The addition of recombinant rat CD38 nullified the inhibitory effect of the diabetic sera. Furthermore, the intracellular cADPR levels in rat islets incubated with the diabetic sera were significantly reduced, and the addition of recombinant CD38 in the medium attenuated the reduction [30], suggesting that the autoantibodies against CD38 inhibited the cADPR-synthesizing enzyme activity of CD38 and that the inhibition of glucose-induced insulin secretion is due to a change in the cADPR concentrations regulated by CD38. When CD38 enzymic activities were measured with the addition of diabetic sera in the enzyme assay mixture, the ADP-ribosyl cyclase activity of CD38 was significantly inhibited (*p* < 0.05 vs. the addition of non-diabetic sera), and the cADPR hydrolase activity of CD38 was instead activated (*p* < 0.05 vs. the addition of non-diabetic sera). These results well explain why the diabetic sera inhibited the glucose-induced insulin secretion from pancreatic β-cells, and the presence of anti-CD38 autoantibodies in diabetic patients is a major cause of impaired glucose-induced insulin secretion, which is frequently found in type 2 diabetes.

Anti-CD38 autoantibodies were later found in Caucasian diabetic patients, and some of their sera showed increased intracellular Ca^2+^ concentrations and glucose-independent insulin secretion from human islets [73,74,75]. In CD38 autoantibodies found in diabetes patients, some autoantibodies show agonistic effects on insulin secretion and others show antagonistic effects. Among the CD38 autoantibodies found in diabetic patients, differences in the effects between Japanese and Caucasian autoantibodies may be explained by the nature of the antibodies and the difference in islet species (rat and human) in the analyses of glucose-induced insulin secretion.

### 6.2. Pathological Significance of the CD38-cADPR System Other Than Diabetes

Although IP_3_ has been thought to be a second messenger for Ca^2+^ mobilization from intracellular stores, cADPR induces Ca^2+^ release from pancreatic islet microsomes but IP_3_ does not, as described above. In the microsomes of the cerebellum, Ca^2+^ release is induced by both cADPR and IP_3_. It is, therefore, apparent that cells can utilize two second-messengers, IP_3_ and cADPR, for Ca^2+^ mobilization, depending on the types of cells as well as differences in cellular conditions, physiological or pathological, performing a variety of cellular functions. Recently, various physiological phenomena in animal, plant, and bacterial cells have been found to utilize this novel signal system [5,19,32,44,76,77,78,79,80,81,82,83,84,85,86,87,88,89,90]. In the pancreatic exocrine acinar cells of CD38 knockout mice, the acetylcholine-induced Ca^2+^ oscillation was greatly reduced or completely disappeared under a physiological concentration of acetylcholine (40 nM–4 µM) [83]. Furthermore, acetylcholine induced cADPR formation in normal (CD38^+/+^) exocrine acinar cells but not in CD38 knockout exocrine acinar cells. The IP_3_ formation was very small in the presence of a physiological concentration of acetylcholine (40 nM–4 µM) and showed no difference between normal and CD38 knockout pancreatic acinar cells. Acetylcholine appears to induce the cADPR formation via the G-protein-coupled CD38 system [79]. In pancreatic islet β-cells, glucose is metabolized to form ATP and increases cADPR in response to glucose stimulation through the CD38-cADPR signal system for insulin secretion. In other cells such as pancreatic acinar cells and neuronal cells, hormones and neurotransmitters regulate the CD38-cADPR-Ca^2+^ signaling system in a receptor-coupled manner, such as in a G-protein-coupled manner, for various types of physiological responses [33,34].

The possible involvement of the CD38-cADPR signal system in cardiovascular abnormalities has been reported. Myocardial hypertrophy was observed in male mice with null mutations of CD38 and FKBP12.6, a cADPR binding protein [84,85]. In addition, the altered stoichiometry of FKBP12.6 versus type 2 RyR as a cause of abnormal Ca^2+^ leak through RyR was reported in heart failure in humans [82]. As diabetic complications in the cardiovascular system are frequently observed, screening of the abnormalities in the CD38-cADPR signal system and its components may provide a clue as to the underlying molecular mechanism. Furthermore, the CD38-cADPR signal was recently revealed to be a regulator of renin expression in juxtaglomerular cells in intermittent hypoxia (IH) [91]. As hypertension is the most common complication in sleep apnea syndrome (SAS) patients, and cells and tissues in SAS patients are exposed to IH, the finding could well explain hypertension in SAS patients.

Jin et al., revealed that adult CD38 knockout female and male mice in CD-1 mouse background showed marked defects in maternal nurturing and social behavior [5]. The plasma level of oxytocin was significantly decreased in CD38 knockout mice and oxytocin injection or lentiviral-vector-mediated delivery of human CD38 in the hypothalamus rescued social memory and maternal care in CD38 knockout mice [5]. In contrast, the introduction of lentiviral-vector mediated delivery of human CD38 Arg140Trp (rs1800561), which had first been found in Japanese type 2 diabetic patients [29], failed to recover the social memory and maternal care. Depolarization-induced oxytocin secretion and Ca^2+^ elevation in oxytocinergic neurohypophysial axon terminals were disrupted in the CD38 knockout mice. These results indicate that CD38 has a key role in neuropeptide release, such as oxytocin release, thereby decisively regulating maternal and social behaviors, and may be a component in neurodevelopmental disorders. The CD38 mutation that caused tryptophan to replace arginine at amino acid residue 140 (R140W; [rs1800561, 4693C > T]) [29] was found not only in Japanese type 2 diabetes patients but also in 0.6–4.6% of the Japanese population and was associated with autism spectrum disorder in a smaller case-control study [92]. In addition, maternal CD38R140W (rs1800561[4693C > T]) polymorphism was reported to be associated with an increased risk of admission to the neonatal intensive care unit due to preterm birth in Nara Medical University, Japan [93]. These reports strongly suggest that the CD38R140W (rs1800561[4693C > T]) polymorphism is a disease-prone genotype although the polymorphism is found to be only in mongoloid subjects.

Most recently, Takeda et al. reported IH, which is a common feature of SAS patient cells, induced the upregulation of renin in juxtaglomerular cells via the upregulation of Cd38 [91]. The IH-induced upregulation of renin was attenuated by the introduction of small interfering RNA for Cd38 or the addition of the cADPR antagonist, 8-bromo-cADPR, in the juxtaglomerular cell culture medium.

## Abbreviations

A-kinase	Cyclic AMP-dependent protein kinase
BST-1	Bone marrow stromal cell antigen 1
cADPR	Cyclic ADP-ribose
cAMP	Cyclic AMP
CaM	Calmodulin
CaM kinase	Ca^2+^/calmodulin-dependent protein kinase
CD38	Cluster of differentiation 38
CD157	Cluster of differentiation 157
CICR	Ca^2+^-induced Ca^2+^ release
EPAC2	Exchange protein activated by cAMP2
FKBP	FK506 binding protein
GIP	Gastric inhibitory polypeptide
GLP-1	Glucagon-like peptide-1
IH	Intermittent hypoxia
IP_3_	Inositol 1,4,5-trisphosphate
NAADP	Nicotinic acid adenine dinucleotide phosphate
RyR	Ryanodine receptor Ca^2+^ channel
SAS	Sleep apnea syndrome

## References

[1] Berridge M.J., Lipp P., Bootman M.D. (2000). The versatility of calcium signalling. Nat. Rev. Mol Cell Biol..

[2] Lee H.C. (2001). Physiological functions of cyclic ADP-ribose and NAADP as calcium messengers. Annu. Rev. Pharmacol. Toxicol..

[3] Guse A.H., Lee H.C. (2008). NAADP: A universal Ca^2+^ trigger. Sci. Signal..

[4] Clapper D.L., Walseth T.F., Dargie P.J., Lee H.C. (1987). Pyridine nucleotide metabolites stimulate calcium release from sea urchin egg microsomes desensitized to inositol trisphosphate. J. Biol. Chem..

[5] Jin D., Liu H.X., Hirai H., Torashima T., Nagai T., Lopatina O., Shnayder N.A., Yamada K., Noda M., Seike T. (2007). CD38 is critical for social behaviour by regulating oxytocin secretion. Nature.

[6] Hellmich M.R., Strumwasser F. (1991). Purification and characterization of a molluscan egg-specific NADase, a second-messenger enzyme. Cell Regul..

[7] Lee H.C., Aarhus R. (1991). ADP-ribosyl cyclase: An enzyme that cyclizes NAD^+^ into a calcium-mobilizing metabolite. Cell Regul..

[8] Glick D.L., Hellmich M.R., Beushausen S., Tempst P., Bayley H., Strumwasser F. (1991). Primary structure of a molluscan egg-specific NADase, a second-messenger enzyme. Cell Regul..

[9] Nata K., Sugimoto T., Tohgo A., Takamura T., Noguchi N., Matsuoka A., Numakunai T., Shikama K., Yonekura H., Takasawa S. (1995). The structure of the *Aplysia kurodai* gene encoding ADP-ribosyl cyclase, a second-messenger enzyme. Gene.

[10] States D.J., Walseth T.F., Lee H.C. (1992). Similarities in amino acid sequences of Aplysia ADP-ribosyl cyclase and human leukocyte antigen CD38. Trends. Biochem. Sci..

[11] Howard M., Grimaldi J.C., Bazan J.F., Lund F.E., Santos-Argumedo L., Parkhouse R.M., Walseth T.F., Lee H.C. (1993). Formation and hydrolysis of cyclic ADP-ribose catalyzed by lymphocyte antigen CD38. Science.

[12] Takasawa S., Tohgo A., Noguchi N., Koguma T., Nata K., Sugimoto T., Yonekura H., Okamoto H. (1993). Synthesis and hydrolysis of cyclic ADP-ribose by human leukocyte antigen CD38 and inhibition of the hydrolysis by ATP. J. Biol. Chem..

[13] Summerhill R.J., Jackson D.G., Galione A. (1993). Human leukocyte antigen CD38 catalyzes the production of cyclic ADP-ribose. FEBS Lett..

[14] Hirata Y., Kimura N., Sato K., Ohsugi Y., Takasawa S., Okamoto H., Ishikawa J., Kaisho T., Ishihara K., Hirano T. (1994). ADP ribosyl cyclase activity of a novel bone marrow stromal cell surface molecule, BST-1. FEBS Lett..

[15] Chini E.N., Chini C.C., Kato I., Takasawa S., Okamoto H. (2002). CD38 is the major enzyme responsible for synthesis of nicotinic acid-adenine dinucleotide phosphate in mammalian tissues. Biochem. J..

[16] Ashcroft F.M., Harrison D.E., Ashcroft S.J.H. (1984). Glucose induced closure of single potassium channels in isolated rat pancreatic β-cells. Nature.

[17] Takasawa S., Nata K., Yonekura H., Okamoto H. (1993). Cyclic ADP-ribose in insulin secretion from pancreatic β cells. Science.

[18] Okamoto H., Takasawa S., Tohgo H. (1995). New aspects of the physiological significance of NAD, poly ADP-ribose and cyclic ADP-ribose. Biochimie.

[19] Okamoto H., Takasawa S., Nata K. (1997). The CD38-cyclic ADP-ribose signalling system in insulin secretion: Molecular basis and clinical implications. Diabetologia.

[20] Koguma T., Takasawa S., Tohgo A., Karasawa T., Furuya Y., Yonekura H., Okamoto H. (1994). Cloning and characterization of cDNA encoding rat ADP-ribosyl cyclase/cyclic ADP-ribose hydrolase (homologue to human CD38) from islets of Langerhans. Biochim. Biophys. Acta.

[21] Tohgo A., Takasawa S., Noguchi N., Koguma T., Nata K., Sugimoto T., Furuya Y., Yonekura H., Okamoto H. (1994). Essential cysteine residues for cyclic ADP-ribose synthesis and hydrolysis by CD38. J. Biol. Chem..

[22] Nakagawara K., Mori M., Takasawa S., Nata K., Takamura T., Berlova A., Tohgo A., Karasawa T., Yonekura H., Takeuchi T. (1995). Assignment of CD38, the gene encoding human leukocyte antigen CD38 (ADP-ribosyl cyclase/cyclic ADP-ribose hydrolase), to chromosome 4p15. Cytogenet. Cell Genet..

[23] Kato I., Takasawa S., Akabane A., Tanaka O., Abe H., Takamura T., Suzuki Y., Nata K., Yonekura H., Yoshimoto T. (1995). Regulatory role of CD38 (ADP-ribosyl cyclase/cyclic ADP-ribose hydrolase) in insulin secretion by glucose in pancreatic β cells. Enhanced insulin secretion in CD38-expressing transgenic mice. J. Biol. Chem..

[24] Takasawa S., Ishida A., Nata K., Nakagawa K., Noguchi N., Tohgo A., Kato I., Yonekura H., Fujisawa H., Okamoto H. (1995). Requirement of calmodulin-dependent protein kinase II in cyclic ADP-ribose-mediated intracellular Ca^2+^ mobilization. J. Biol. Chem..

[25] Noguchi N., Takasawa S., Nata K., Tohgo A., Kato I., Ikehata F., Yonekura H., Okamoto H. (1997). Cyclic ADP-ribose binds to FK506-binding protein 12.6 to release Ca^2+^ from islet microsomes. J. Biol. Chem..

[26] Tohgo A., Munakata H., Takasawa S., Nata K., Akiyama T., Hayashi N., Okamoto H. (1997). Lysine 129 of CD38 (ADP-ribosyl cyclase/cyclic ADP-ribose hydrolase) participates in the binding of ATP to inhibit the cyclic ADP-ribose hydrolase. J. Biol. Chem..

[27] Nata K., Takamura T., Karasawa T., Kumagai T., Hashioka W., Tohgo A., Yonekura H., Takasawa S., Nakamura S., Okamoto H. (1997). Human gene encoding CD38 (ADP-ribosyl cyclase/cyclic ADP-ribose hydrolase): Organization, nucleotide sequence and alternative splicing. Gene.

[28] Takasawa S., Akiyama T., Nata K., Kuroki M., Tohgo A., Noguchi N., Kobayashi S., Kato I., Katada T., Okamoto H. (1998). Cyclic ADP-ribose and inositol 1,4,5-trisphosphate as alternate second messengers for intracellular Ca^2+^ mobilization in normal and diabetic β-cells. J. Biol. Chem..

[29] Yagui K., Shimada F., Miura M., Hashimoto N., Suzuki Y., Tokuyama Y., Nata K., Tohgo A., Ikehata F., Takasawa S. (1998). A missense mutation in the CD38 gene, a novel factor for insulin secretion: Association with Type II diabetes mellitus in Japanese subjects and evidence of abnormal function when expressed in vitro. Diabetologia.

[30] Ikehata F., Satoh J., Nata K., Tohgo A., Nakazawa T., Kato I., Kobayashi S., Akiyama T., Takasawa S., Toyota T. (1998). Autoantibodies against CD38 (ADP-ribosyl cyclase/cyclic ADP-ribose hydrolase) that impair glucose-induced insulin secretion in noninsulin- dependent diabetes patients. J. Clin. Investig..

[31] Kato I., Yamamoto Y., Fujimura M., Noguchi N., Takasawa S., Okamoto H. (1999). CD38 disruption impairs glucose-induced increases in cyclic ADP-ribose, [Ca^2+^]i, and insulin secretion. J. Biol. Chem..

[32] Okamoto H., Takasawa S., Nata K., Kato I., Tohgo A., Noguchi N. (2000). Physiological and pathological significance of the CD38-cyclic ADP-ribose signaling system. Chem. Immunol..

[33] Takasawa S., Okamoto H. (2002). Pancreatic β-cell death, regeneration and insulin secretion: Roles of poly(ADP-ribose) polymerase and cyclic ADP-ribose. Int. J. Diabetes Res..

[34] Okamoto H., Takasawa S. (2002). Recent advances in the Okamoto model: The CD38-cyclic ADP-ribose signal system and the regenerating gene protein (Reg)-Reg receptor system in β-cells. Diabetes.

[35] Islam M.S., Larsson O., Berggren P.O., Takasawa S., Nata K., Yonekura H., Okamoto H., Galione A. (1993). Cyclic ADP-ribose in β cells. Science.

[36] Rutter G.A., Theler J.-M., Wollheim C.B. (1994). Ca^2+^ stores in insulin-secreting cells: Lack of the effect of cADP ribose. Cell Calcium.

[37] Webb D.-L., Islam M.S., Efanov A.M., Brown G., Köhler M., Larsson O., Berggren P.-O. (1996). Insulin exocytosis and glucose-mediated increase in cytoplasmic free Ca^2+^ concentration in the pancreatic β-cell are independent of cyclic ADP-ribose. J. Biol. Chem..

[38] Islam M.S., Berggren P.O. (1997). Cyclic ADP-ribose and the pancreatic beta cell: Where do we stand?. Diabetologia.

[39] Malaisse W.J., Kanda Y., Inageda K., Scruel O., Sener A., Katada T. (1997). Cyclic ADP-ribose measurements in rat pancreatic islets. Biochem. Biophys. Res. Commun..

[40] Scruel O., Wada T., Kontani K., Sener A., Katada T., Malaisse W.J. (1998). Effects of D-glucose and starvation upon the cyclic ADP-ribose content of rat pancreatic islets. Biochem. Mol. Biol. Int..

[41] An N.H., Han M.K., Um C., Park B.H., Park B.J., Kim H.K., Kim U.H. (2001). Significance of ecto-cyclase activity of CD38 in insulin secretion of mouse pancreatic islet cells. Biochem. Biophys. Res. Commun..

[42] Varadi A., Rutter G.A. (2002). Dynamic imaging of endoplasmic reticulum Ca^2+^ concentration in insulin-secreting MIN6 cells using recombinant targeted cameleons: Roles of sarco(endo)plasmic reticulum Ca^2+^-ATPase (SERCA)-2 and ryanodine receptors. Diabetes.

[43] Mitchell K.J., Pinton P., Varadi A., Tacchetti C., Ainscow E.K., Pozzan T., Rizzuto R., Rutter G.A. (2001). Dense core secretory vesicles revealed as a dynamic Ca^2+^ store in neuroendocrine cells with a vesicle-associated membrane protein aequorin chimaera. J. Cell Biol..

[44] Ikeda T., Takasawa S., Noguchi N., Nata K., Yamauchi A., Takahashi I., Yoshikawa T., Sugawara A., Yonekura H., Okamoto H. (2012). Identification of a major enzyme for the synthesis and hydrolysis of cyclic ADP-ribose in amphibian cells and evolutional conservation of the enzyme from human to invertebrate. Mol. Cell. Biochem..

[45] Zocchi E., Franco L., Guida L., Benatti U., Bagellesi A., Malavasi F., Lee H.C., De Flora A. (1993). A single protein immunologically identified as CD38 displays NAD^+^ glycohydrolase, ADP-ribosyl cyclase and cyclic ADP-ribose hydrolase activities at the outer surface of human erythrocytes. Biochem. Biophys. Res. Commun..

[46] Munshi C., Thiel D.J., Mathews I.I., Aarhus R., Walseth T.F., Lee H.C. (1999). Characterization of the active site of ADP-ribosyl cyclase. J. Biol. Chem..

[47] Okamoto H., Takasawa S., Creighton T.E. (2002). CD38. Encyclopedia of Molecular Medicine.

[48] Ferrero E., Lo Buono N., Morone S., Parrotta R., Mancini C., Brusco A., Giancomino A., Augeri S., Rosal-Vela A., García-Rodríguez S. (2017). Human canonical CD157/Bst1 is an alternatively spliced isoform masking a previously unidentified primate-specific exon included in a novel transcript. Sci. Rep..

[49] Kaisho T., Ishikawa J., Oritani K., Inazawa J., Muraoka O., Ochi T., Hirano T. (1994). BST-1, a surface molecule of bone marrow stromal cell lines that facilitates pre-B-cell growth. Proc. Natl. Acad. Sci. USA.

[50] Muraoka O., Tanaka H., Itoh M., Ishihara K., Hirano T. (1996). Genomic structure of human BST-1. Immunol. Lett..

[51] Dong C., Willerford D., Alt F.W., Cooper M.D. (1996). Genomic organization and chromosomal localization of the mouse BP3 gene, a member of the CD38/ADP-ribosy cyclase family. Immunogenetics.

[52] Furuya Y., Takasawa S., Yonekura H., Tanaka T., Takehara J., Okamoto H. (1995). Cloning of a cDNA encoding rat bone marrow stromal cell antigen 1 (BST-1) from the islets of Langerhans. Gene.

[53] Kajimoto Y., Miyagawa J., Ishihara K., Okuyama Y., Fujitani Y., Itoh M., Yoshida H., Kaisho T., Matsuoka T., Watada H. (1996). Pancreatic islets cells express BST-1, a CD38-like surface molecule having ADP-ribosyl cyclase activity. Biochem. Biophys. Res. Commun..

[54] Itoh M., Ishihara K., Hiroi T., Lee B.O., Maeda H., Iijima H., Yanagita M., Kiyono H., Hirano H. (1998). Deletion of bone marrow stromal cell antigen-1 (CD157) gene impaired systemic thymus independent-2 antigen-induced IgG3 and mucosal TD antigen-elicited IgA responses. J. Immunol..

[55] Jackson D.G., Bell J.I. (1990). Isolation of a cDNA encoding the human CD38 (T10) molecule, a cell surface glycoprotein with an unusual discontinuous pattern of expression during lymphocyte differentiation. J. Immunol..

[56] Harada N., Santos-Arugumedo L., Chang R., Grimaldi J.C., Lund F.E., Brannan C.I., Copeland N.G., Jenkins N.A., Heath A.W., Parkhouse R.M. (1993). Expression cloning of a cDNA encoding a novel murine B cell activation marker. Homology to human CD38. J. Immunol..

[57] Itoh M., Ishihara K., Tomizawa H., Tanaka H., Kobune Y., Ishikawa J., Kaisho T., Hirano T. (1994). Molecular cloning of murine BST-1 having homology with CD38 and *Aplysia* ADP-ribosyl cyclase. Biochem. Biophys. Res. Commun..

[58] Churamani D., Boulware M.J., Geach T.J., Martin A.C.R., Moy G.W., Su Y.-H., Vacquier V.D., Marchant J.S., Dale L., Patel S. (2007). Molecular characterization of a novel intracellular ADP-ribosyl cyclase. PLoS ONE.

[59] Goodrich S.P., Muller-Steffner M., Osman A., Moutin M.-J., Kusser K., Roberts A., Woodland D.L., Randall T.D., Kellenberger E., LoVerde P.T. (2005). Production of calcium-mobilizing metabolites by a novel member of the ADP-ribosyl cyclase family expressed in *Schistosoma mansoni*. Biochemistry.

[60] Kelu J.J., Web S.E., Galione A., Miller A.L. (2019). Characterization of ADP-ribosyl cyclase 1-like (ARC1-like) activity and NAADP signaling during slow muscle cell development in zebrafish embryos. Dev. Biol..

[61] Noguchi N., Yoshikawa T., Ikeda T., Takahashi I., Shervani N.J., Uruno A., Yamauchi A., Nata K., Takasawa S., Okamoto H. (2008). FKBP12.6 disruption impairs glucose-induced insulin secretion. Biochem. Biophys. Res. Commun..

[62] Ashcroft F.M., Ashcroft S.J.H. (1992). Insulin: Molecular Biology to Pathology.

[63] Galione A. (1993). Cyclic ADP-ribose: A new way to control calcium. Science.

[64] Takasawa S., Kuroki M., Nata K., Noguchi N., Ikeda T., Yamauchi A., Ota H., Itaya-Hironaka A., Sakuramoto-Tsuchida S., Takahashi I. (2010). A novel ryanodine receptor expressed in pancreatic islets by alternative splicing from type 2 ryanodine receptor gene. Biochem. Biophys. Res. Commun..

[65] Pirsch J.D., Miller J., Deierhoi M.H., Vincenti F., Filo R.S. (1997). A comparison of tacrolimus (FK506) and cyclosporine for immunosuppression after cadaveric renal transplantation: FK506 Kidney Transplant Study Group. Transplantation.

[66] Seino Y., Fukushima M., Yabe D. (2010). GIP and GLP-1, the two incretin hormones: Similarities and differences. J. Diabetes Investig..

[67] Kim B.J., Park K.H., Yim C.Y., Takasawa S., Okamoto H., Im M.J., Kim U.H. (2008). Generation of nicotinic acid adenine dinucleotide phosphate and cyclic ADP-ribose by glucagon-like peptide-1 evokes Ca^2+^ signal that is essential for insulin secretion in mouse pancreatic islets. Diabetes.

[68] Okamoto H., Takasawa S., Yamamoto Y. (2017). From insulin synthesis to secretion: Alternative splicing of type 2 ryanodine receptor gene is essential for insulin secretion in pancreatic β cells. Int. J. Biochem. Cell Biol..

[69] Makino M., Itaya-Hironaka A., Yamauchi A., Sakuramoto-Tsuchida S., Takasawa S. (2020). Tissue-specific alternative splicing of type 2 ryanodine receptor gene affects insulin biosynthesis in pancreatic β cells. Diabetologia.

[70] Mount S.M. (1982). A catalog of splice junction sequences. Nucleic Acids Res..

[71] Nakai K., Sakamoto H. (1994). Construction of a novel database containing aberrant splicing mutations of mammalian genes. Gene.

[72] Sakai N., Santamarina-Fojo S., Yamashita S., Matsuzawa Y., Brewer H.B. (1996). Exon 10 skipping caused by intron 10 splice donor site mutation in cholesterol ester transfer protein gene results in abnormal downstream splice site selection. J. Lipid Res..

[73] Pupilli C., Giannini S., Marchetti P., Lupi R., Antonelli A., Malavasi F., Takasawa S., Okamoto H., Ferrannini E. (1999). Autoantibodies to CD38 (ADP-ribosyl cyclase/cyclic ADP-ribose hydrolase) in Caucasian patients with diabetes: Effects on insulin release from human islets. Diabetes.

[74] Mallone R., Ortolan E., Baj G., Funaro A., Giunti S., Lillaz E., Saccucci F., Cassader M., Cavallo-Perin P., Malavasi F. (2001). Autoantibody response to CD38 in Caucasian patients with type 1 and type 2 diabetes: Immunological and genetic characterization. Diabetes.

[75] Antonelli A., Baj G., Marchetti P., Fallahi P., Surico N., Pupilli C., Malavasi F., Ferrannini E. (2001). Human anto-CD38 autoantibodies raise intracellular calcium and stimulate insulin release in human pancreatic islets. Diabetes.

[76] Hua S.Y., Tokimasa T., Takasawa S., Furuya Y., Nohmi M., Okamoto H., Kuba K. (1994). Cyclic ADP-ribose modulates Ca^2+^ release channels for activation by physiological Ca^2+^ entry in bullfrog sympathetic neurons. Neuron.

[77] Karasawa T., Takasawa S., Yamakawa K., Yonekura H., Okamoto H., Nakamura S. (1995). NAD^+^-glycohydrolase from *Streptococcus pyogenes* shows cyclic ADP-ribose forming activity. FEMS Microbiol. Lett..

[78] Ebihara S., Sasaki T., Hida W., Kikuchi Y., Ohshiro T., Shimura S., Takasawa S., Okamoto H., Nishiyama A., Akaike N. (1997). Role of cyclic ADP-ribose in ATP-activated potassium currents in alveolar macrophages. J. Biol. Chem..

[79] Higashida H., Yokoyama S., Hashii M., Taketo M., Higashida M., Takayasu T., Ohshima T., Takasawa S., Okamoto H., Noda M. (1997). Muscarinic receptor-mediated dual regulation of ADP-ribosyl cyclase in NG108-15 neuronal cell membranes. J. Biol. Chem..

[80] Wu Y., Kuzma J., Maréchal E., Graeff R., Lee H.C., Foster R., Chua N.-H. (1997). Abscisic acid signalling through cyclic ADP-ribose in plats. Science.

[81] Leckie C.P., McAinsh M.R., Allen G.J., Sanders D., Hetherington A.M. (1998). Abscisic acid-induced stomatal closure mediated by cyclic ADP-ribose. Proc. Natl. Acad. Sci. USA.

[82] Yano M., Ono K., Ohkusa T., Suetsugu M., Kohno M., Hisaoka T., Kobayashi S., Hisamatsu Y., Yamamoto T., Kohno M. (2000). Altered stoichiometry of FKBP12.6 versus ryanodine receptor as a cause of abnormal Ca^2+^ leak through ryanodine receptor in heart failure. Circilation.

[83] Fukushi Y., Kato I., Takasawa S., Sasaki T., Ong B.H., Sato M., Ohsaga A., Sato K., Shirato K., Okamoto H. (2001). Identification of cyclic ADP-ribose-dependent mechanisms in pancreatic muscarinic Ca^2+^ signaling using CD38 knockout mice. J. Biol. Chem..

[84] Xin H.-B., Senbonmatsu T., Cheng D.-S., Wang Y.-X., Copello J.A., Ji G.-J., Collier M.L., Deng Y.-X., Jeyakumar L.H., Magnuson M.A. (2002). Oestrogen protects FKBP12.6 null mice from cardiac hypertrophy. Nature.

[85] Takahashi J., Kagaya Y., Kato I., Ohta J., Isoyama S., Miura M., Sugai Y., Hirose M., Wakayama Y., Ninomiya M. (2003). Deficit of CD38/cyclic ADP-ribose is differentially compensated in hearts by gender. Biochem. Biophys. Res. Commun..

[86] Mitsui-Saito M., Kato I., Takasawa S., Okamoto H., Yanagisawa T. (2003). CD38 gene disruption inhibits the contraction induced by α-adrenoceptor stimulation in mouse aorta. J. Vet. Med. Sci..

[87] Sasamori K., Sasaki T., Takasawa S., Tamada T., Nara M., Irokawa T., Shimura S., Shirato K., Hattori T. (2004). Cyclic ADP-ribose, a putative Ca^2+^-mobilizing second messenger, operates in submucosal gland acinar cells. Am. J. Physiol. Lung Cell Mol. Physiol..

[88] Fellner S.K., Parker L. (2005). Endothelin-1, superoxide and adeninediphosphate ribose cyclase in shark vascular smooth muscle. J. Exp. Biol..

[89] Dodd A.N., Gardner M.J., Hotta C.T., Hubbard K.E., Delchau N., Love J., Assie J.M., Robertson F.C., Jakobsen M.K., Gonçalves J. (2007). The Arabidopsis circadian clock incorporates a cADPR-based feedback loop. Science.

[90] Ota H., Tamaki S., Itaya-Hironaka A., Yamauchi A., Sakuramoto-Tsuchida S., Morioka T., Takasawa S., Kimura H. (2012). Attenuation of glucose-induced insulin secretion by intermittent hypoxia via down-regulation of CD38. Life Sci..

[91] Takeda Y., Itaya-Hironaka A., Yamauchi A., Makino M., Sakuramoto-Tsuchida S., Ota H., Kawaguchi R., Takasawa S. (2021). Intermittent hypoxia upregulates the *Renin* and *Cd38* mRNAs in renin-producing cells via the downregulation of miR-203. Int. J. Mol. Sci..

[92] Munesue T., Yokoyama S., Nakamura K., Anitha A., Yamada K., Hayashi K., Asaka T., Liu H.X., Jin D., Koizumi K. (2010). Two genetic variants of CD38 in subjects with autism spectrum disorder and controls. Neurosci. Res..

[93] Enami N., Itaya-Hironaka A., Yamauchi A., Sakuramoto-Tsuchida S., Takasawa S., Takahashi Y. (2015). The CD38 genotype (rs1800561 (4693C>T): R140W) is associated with an increased risk of admission to the neonatal intensive care unit. Early Hum. Dev..

